# A dataset of annotated African plum images from Cameroon for AI-based quality assessment

**DOI:** 10.1016/j.dib.2025.111351

**Published:** 2025-01-30

**Authors:** Arnaud Nguembang Fadja, Armel Gabin Fameni Tagni, Sain Rigobert Che, Marcellin Atemkeng

**Affiliations:** a Department of Engineering, University of Ferrara, Italy; b College of Technology, University of Buea, PO Box 63, Buea, South West Region, Cameroon; c African Institute for Mathematical Sciences, Cameroon; d Department of Mathematics, Rhodes University, South Africa

**Keywords:** African plum, Safou, Agricultural dataset, Fruit quality assessment, Image classification, Object detection, Computer vision, Cameroon

## Abstract

This paper presents a dataset of 4507 annotated images of African plums collected across diverse regions in Cameroon, marking the first dataset specifically designed for AI-driven quality assessment of this fruit. The dataset is categorized into six quality grades: unaffected, bruised, cracked, rotten, spotted, and unripe. These categories represent varying degrees of plum quality, from optimal condition to various defects and ripeness levels. Captured under natural lighting using a consistent smartphone setup, the images were meticulously labeled by agricultural experts, ensuring high annotation accuracy. This resource is valuable for developing and testing computer vision, deep learning-based recognition systems and object detection models in agriculture, enabling automated evaluation of plum quality for commercialization. By offering a comprehensive, culturally relevant dataset focused on a traditionally underrepresented crop, this work supports advancements in precision agriculture, particularly in developing regions. Potential applications include AI-based tools for real-time sorting, defect detection, and quality assurance in the supply chain.

Specifications Table

**Table:** 

Subject	Computer Science, Agriculture
Specific subject area	AI for Agricultural Product Quality Assessment
Type of data	Image (JPEG/PNG)
Data collection	The dataset is about the collection of plum images. It includes images of degraded and fresh plums collected in the field over the range of 2 years from January 2021 to February 2023. The images were captured using the standard settings of a Tecno Camon 12 mobile phone, ensuring consistency and reliability throughout the process. The camera was configured with a fixed aperture, producing sharp and clear images. A resolution of 16MP was utilized to capture high-quality details, while the capture mode, ISO sensitivity, and shutter speed were set to automatic to adapt seamlessly to varying lighting conditions. Image stabilization was enabled to minimize blurring, and the flash was kept off to maintain natural lighting. Additionally, the AI camera filter was activated to enhance image quality, and face detection was employed as the focus mode to ensure accurate subject framing and clarity. This setup provided an efficient and standardized approach to capturing the dataset. The dataset comprises images collected from various regions across Cameroon, with specific counts as follows: 260 images were captured in Limbe and 900 in Buea, both located in the South West region. In the Littoral region, 2255 images were collected in Douala. The Center region contributed 210 images from Yaounde and another 210 from Ayos. Additionally, 202 images were captured in Nguiwa Yangamo in the East region, 170 images in Bafoussam in the West region, and 300 images in Ngaoundere in the Adamawa region. This diverse geographic distribution provides a broad representation of the visual data across multiple regions of the country. They were subsequently annotated with quality grades by agricultural experts.
Data source location	City: limbe Longitude and longitude: 9°11′43.60″E and 4° 1′20.88″N City: Buea Longitude and longitude: 9°15′47.61″E and 4° 9′21.48″N City:Douala Longitude and longitude: 9°46′4.33″E and 4° 3′3.80″N City: Yaoundé Longitude and longitude: 11°31′12.80″E and 3°51′42.07″N City: Bafoussam Longitude and longitude: 10°25′37.68″E and 5°28′53.61″N City: Ayos Longitude and longitude: 12°31′36.58″E and 3°54′20.36″N City: Nguiwa yangamo Longitude and longitude: 14° 2′35.10″E and 4°58′47.78″N City: Ngaoundéré Longitude and longitude: 13°34′6.01″E and 7°19′50.67″N Country: Cameroon
Data accessibility	Repository name: African Plums Dataset Data identification number: 10.34740/kaggle/dsv/9694239 Direct URL to data: https://www.kaggle.com/dsv/9694239 The African Plums Datasetis available on Kaggle at this link. To access it, users need a Kaggle account and can either download the files directly from the webpage or use the Kaggle API with the command *kaggle datasets download -*d *arnaudfadja/african-plums-quality-and-defect-assessment-data*. For API access, ensure the *kaggle.json* token is set up correctly. If you use this dataset, please cite it using the provided BibTeX entry to give proper credit and maintain accessibility for other researchers.
Related research article	In their [1], the authors examined the development and ripening of African plums over two consecutive years, using data collected from 16 trees across four production sites in Cameroon. They correlated detailed climatic data (rainfall, temperature, and humidity) with morphological parameters (fruit size, weight, and color) to define and analyze the phenological stages and their durations. For the experiments, 10 labeled fruits were randomly selected and collected from each tree at two-week intervals throughout the fruit development period.

## 1 Value of the Data

• This dataset provides the first comprehensive collection of annotated African plum images, filling a significant gap in AI and agricultural research.
• It supports the development of AI models for fruit quality assessment, a critical component in improving agricultural productivity and food security in regions where African plums are a staple.
• By providing a structured dataset specifically for African plums, researchers can leverage popular AI models such as YOLO and Faster R-CNN to build and refine object detection, defect classification, and quality assessment systems. These models, when trained on this data, can enhance agricultural productivity and streamline quality control processes, promoting advancements in precision agriculture and contributing valuable insights into the region's unique agricultural challenges and opportunities
• By making the dataset publicly available, it encourages further research into underrepresented crops in AI, fostering innovation in agricultural technologies tailored for developing regions.
• The annotated quality grades provide a benchmark for training and evaluating machine learning models in a real-world agricultural context.

## 2 Background

**Fig. 1:**
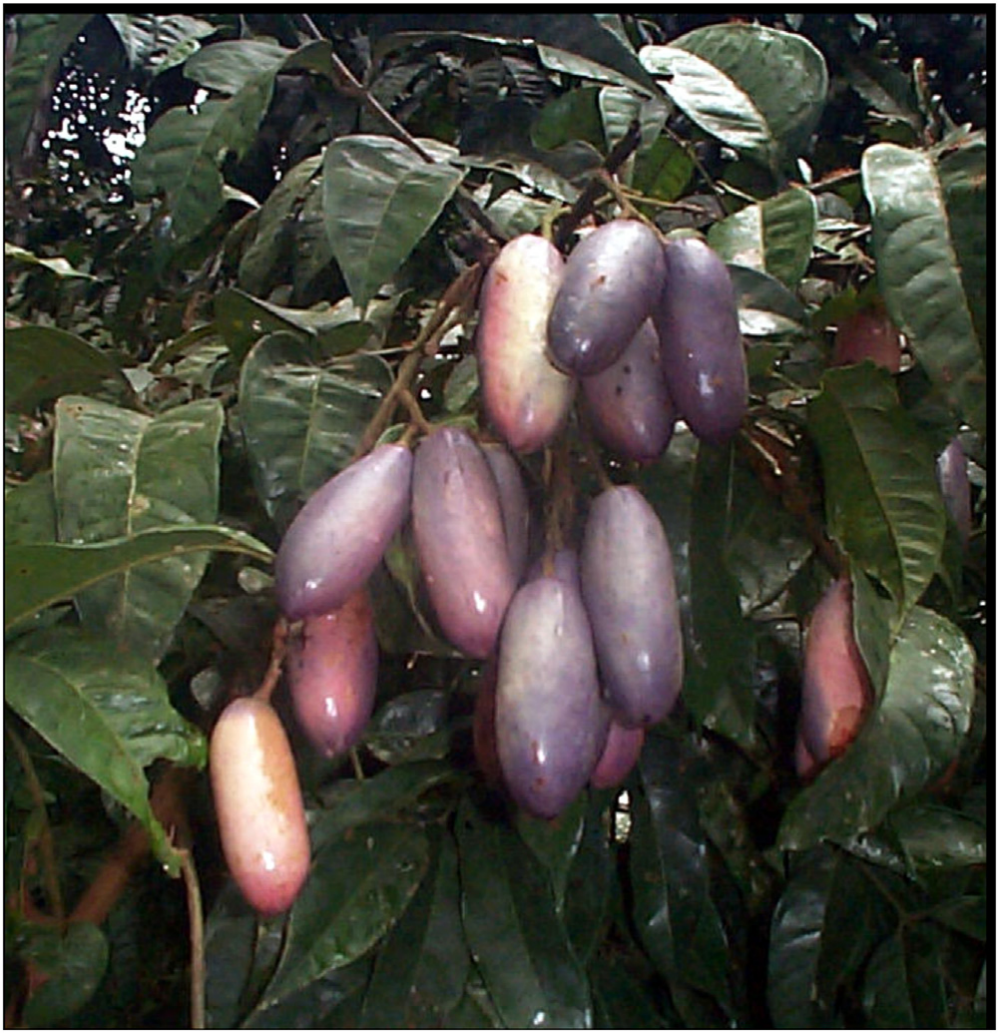
Sample images illustrating various African plum categories [18].

The African plum [[2], [3], [4], [5]] Fig. 1, is a vital agricultural product in West and Central Africa, lacks representation in AI research, particularly in the context of quality assessment. Given its cultural and economic importance, especially in regions like Cameroon, there is a need for reliable, AI-based quality assessment tools. This dataset was compiled to support the development of such tools by providing a diverse and well-annotated collection of images, facilitating the training and evaluation of various machine learning models.

AI-driven techniques for fruit quality assessment have seen substantial growth in recent years, with various datasets and studies focused on specific fruits such as apples, tomatoes, and bananas [[6], [7], [8]]. For example, Kaggle hosts several fruit datasets for object detection and classification, including a comprehensive apple quality dataset aimed at detecting ripeness and defects using machine learning models like YOLO and Faster R-CNN [9,10]. Similarly, a tomato ripeness classification dataset utilised colour and texture features to train convolutional neural networks for quality assessment [11,12].

While these datasets have contributed significantly to AI research in agriculture, African plums, despite their importance in West and Central Africa, remain underrepresented in the current literature [3,4]. Previous studies, [[13], [14], [15], [16]], have not provided sufficient data for developing robust AI models tailored to the unique characteristics of this fruit. Our dataset, [2], is intended to fill this gap by offering annotated images of African plums with quality labels, which can be used to develop machine learning models for quality assessment, object detection, and classification tasks.

Moreover, these datasets are tailored to crops with different morphological characteristics and market relevance. By contrast, our dataset uniquely captures the diverse conditions and challenges specific to African plums, including variations in size, shape, color, and defects, along with imaging conditions from multiple agro-ecological regions. This distinct focus positions our dataset as a critical resource for advancing research and developing AI solutions tailored to African plums, with potential applications in agriculture, supply chain optimization, and food security.

This dataset also contributes to the growing field of AI for precision agriculture, particularly in developing regions where access to cutting-edge technology is limited. By providing a publicly available, annotated dataset focused on a culturally and economically important fruit, this research fosters the development of AI tools that can support agricultural productivity and sustainability in underrepresented regions [17].

## 3 Data Description

The dataset consists of 4507 high-resolution images of African plums collected from three agro-ecological regions in Cameroon. The images capture a wide range of environmental conditions and plum characteristics, providing a diverse dataset for research in computer vision and AI.

**Contents and Structure:**

• **Image Collection:** The dataset consists of images captured under varying lighting conditions and at different times of the day, highlighting plums with diverse sizes, shapes, colors, and defect types. Data collection occurred across multiple locations during sunny days between 8:00 a.m. and 4:00 p.m., ensuring a representative sample of environmental variability. Table 1 provides a detailed distribution of images based on lighting conditions and collection sites. The size of the plums in the dataset ranges from very small (approximately 4 cm in diameter) to medium-sized (up to 10 cm in diameter).

**Table 1:** Image distribution by light condition and location.

Locations	Number of Images	Light Condition
Yaoundé	210	8 am sunlight in the morning
Limbe + Buea + NguiwaYangamo	1362	9 am sunlight in the morning
Douala + Bafoussam	2425	1 pm sunlight in the morning
Ayos + Ngaoundéré	510	4 pm sunlight in the afternoon
• **Annotation:** Each image is assigned a label that defines its category: unaffected (for good-quality plums), unripe and spotted, bruised, cracked, rotten as defective plums, see Fig. 2. Annotation has been carefully conducted and verified for consistency and accuracy. These categories are described as follows:
  ○ **Unaffected:** Plums in optimal condition with no visible defects. This category is ideal for market sale as it represents high-quality produce with the best appearance and shelf life.
  ○ **Unripe:** Plums that have not yet reached full ripeness, typically firmer and less flavorful. While not yet ready for immediate consumption, these plums can be sold for buyers who prefer to ripen fruit at home.
  ○ **Spotted:** Plums with noticeable spots that may be caused by fungal infections, insect damage, or other minor surface defects. While spots might not always affect the taste, they can reduce consumer appeal.
  ○ **Cracked:** Plums with splits or cracks in the skin. Cracks typically result from environmental factors like excessive moisture or uneven growth, which can make the fruit more susceptible to rapid spoilage.
  ○ **Bruised:** Plums that show signs of physical damage, often characterized by dark, softened areas. Bruising can occur during handling or transport, affecting both visual appeal and shelf life.
  ○ **Rotten:** Plums that display visible decay, often accompanied by changes in color, texture, and odor. Rotten plums are generally unsuitable for sale due to quality and health concerns.

**Fig. 2:**
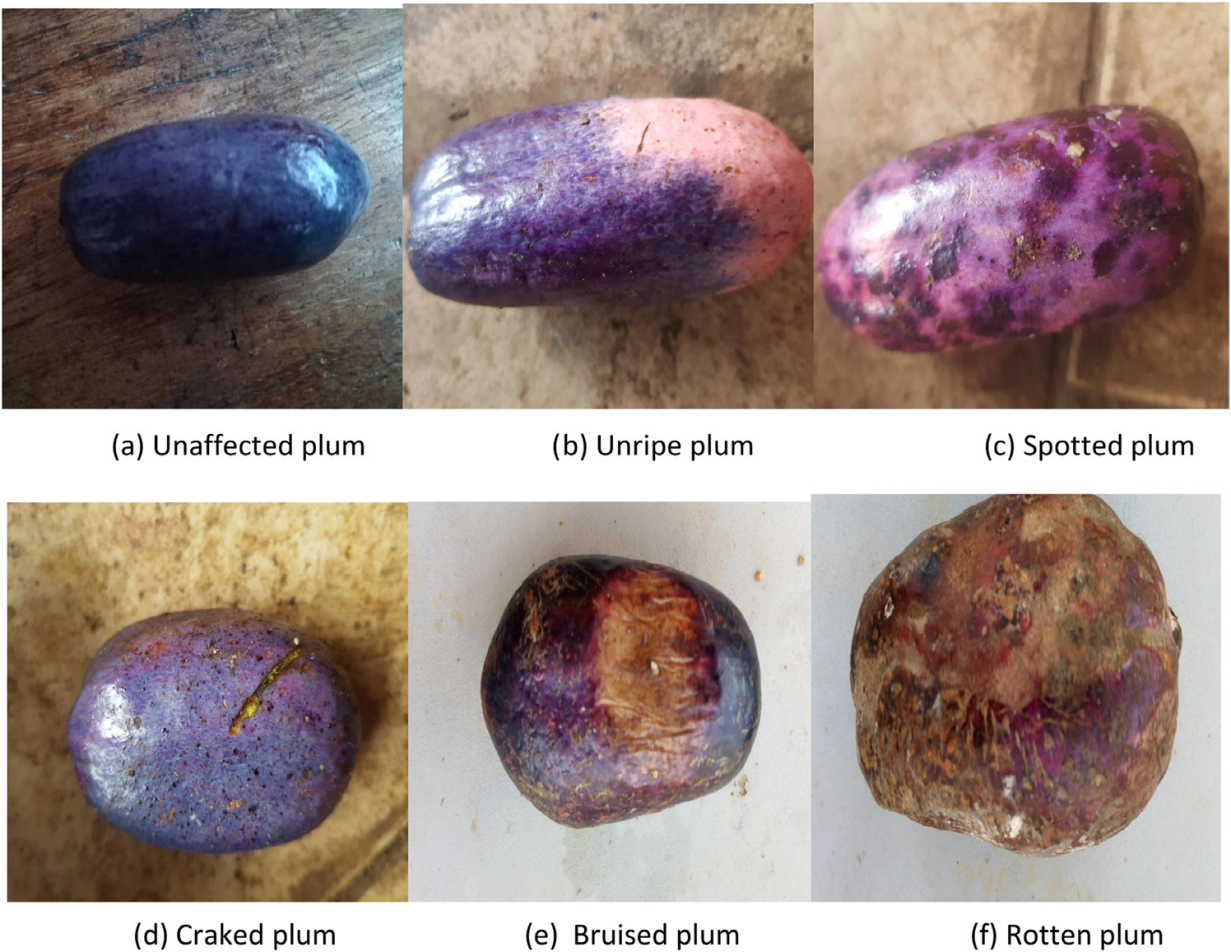
Sample images illustrating various African plum categories.

Organizing plums into these six distinct categories can significantly streamline and enhance the selling process. This targeted approach enables sellers to better meet the demands of various market segments. For example, top-quality, unaffected plums can be sold at premium prices to discerning consumers or high-end retailers, while unripe, cracked, or spotted plums may be offered at standard prices to appeal to general buyers. Categories such as bruised or rotten plums can be efficiently redirected to processing markets, such as oil production, rather than being wasted. This structured classification optimizes resource use, reduces waste, and ensures that each product type meets specific consumer needs, ultimately boosting profitability and customer satisfaction throughout the supply chain.

**Fig. 3:**
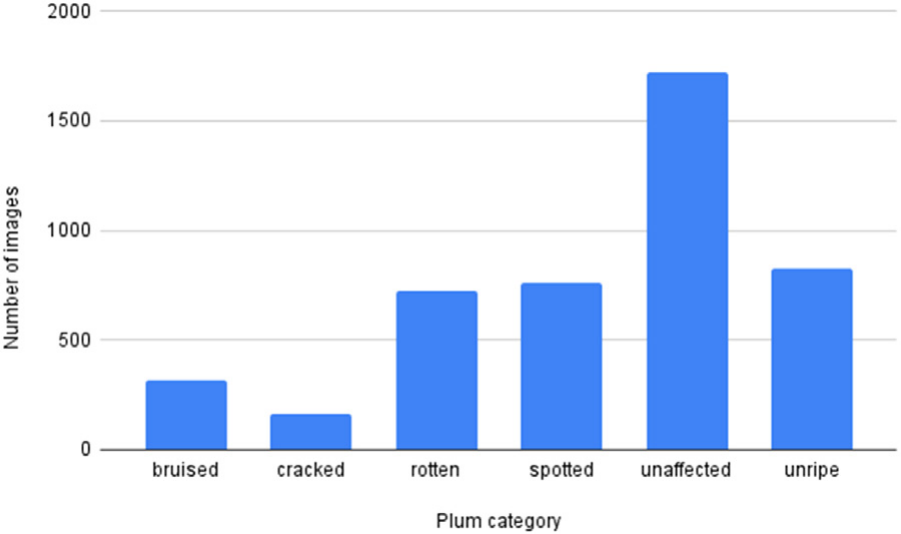
Distribution of the African Plums Dataset.

• **Organisation:** The African Plums dataset is organized into a main folder, "african_plums," containing six subfolders, each representing a defect type distributed as follows, see Fig. 3: unaffected (1721 images), unripe (826 images), spotted (759 images), cracked (162 images),bruised (319 images) and rotten (720 images). Additionally, a CSV file, "plums_data.csv," provides details for each image with three columns: Image ID (formatted as <Defect Type>_plum_<number>), Label (either "defective" for bruised, cracked, rotten, and spotted, "unaffected" for good-quality plums, or "unripe"), and Defect Type, specifying the particular defect.

This dataset is designed to serve as a resource for researchers developing models for automated fruit quality assessment, offering a comprehensive and well-structured collection of annotated images.

## 4 Experimental Design, Materials and Methods

**Data Collection Process:**

• **Geographical Diversity:** Images were captured across three distinct agro-ecological regions in Cameroon, ensuring a broad representation of environmental conditions that affect plum appearance. These specific locations were selected to capture a range of environmental conditions and agricultural practices across Cameroon, ensuring that the dataset reflects the diverse factors affecting plum quality in different regions.
• **Challenges**: During data collection, we encountered several challenges that highlighted the complexities of field-based agricultural research in Cameroon. One significant hurdle was communicating the purpose and value of the research to local farmers, as the concept of data collection for AI-based quality assessment was often unfamiliar. In many instances, to obtain the necessary images, we had to purchase plums ourselves, as some farmers were hesitant to contribute their produce without fully understanding the research's intended application. Additionally, accessibility to remote farming areas posed logistical difficulties, particularly due to poor road conditions and seasonal rainfall, which often made travel arduous and limited the availability of certain plum varieties. These challenges underscore the importance of adaptive strategies and local engagement in the collection of agricultural datasets in developing regions.
• **Capture Techniques:** The images in this dataset were captured using a Tecno Camon 12 mobile phone with its standard settings optimized for consistency and quality. The fixed phone settings included a 16 MP resolution, automatic capture mode, automatic ISO sensitivity, automatic shutter speed, and enabled image stabilization. The flash was kept off, and the AI camera filter option was utilized to enhance image processing. Additionally, the focus mode was set to face detection, ensuring sharpness and clarity in each shot. Using these settings, 3 to 4 perspectives of each plum were captured, leveraging the natural backgrounds available at the diverse collection sites. This approach aimed to reflect the variability of real-world environments while maintaining high-quality image standards.

**Annotation Methodology:**

• **Expert Involvement:** Agricultural experts annotated the images, categorising them based on visual quality. A second expert reviewed the annotations to ensure accuracy. Experts categorized the plums based on specific visual criteria: for example, *spotted plums* exhibited surface blemishes caused by fungal infections or insect damage, while *bruised plums* were identified by darker, softened areas indicative of physical impact, ensuring a consistent and reliable classification standard across the dataset.

**Dataset Preparation:**

• **Data Splitting:** The dataset was divided into 6 folders, each representing a category
• **Format and Accessibility:** The dataset is formatted for compatibility with standard machine learning frameworks, with a clear folder structure for images and annotations. This setup facilitates straightforward integration into research projects focused on agricultural AI applications.

### 4.1 African plum quality detection

**Fig. 4:**
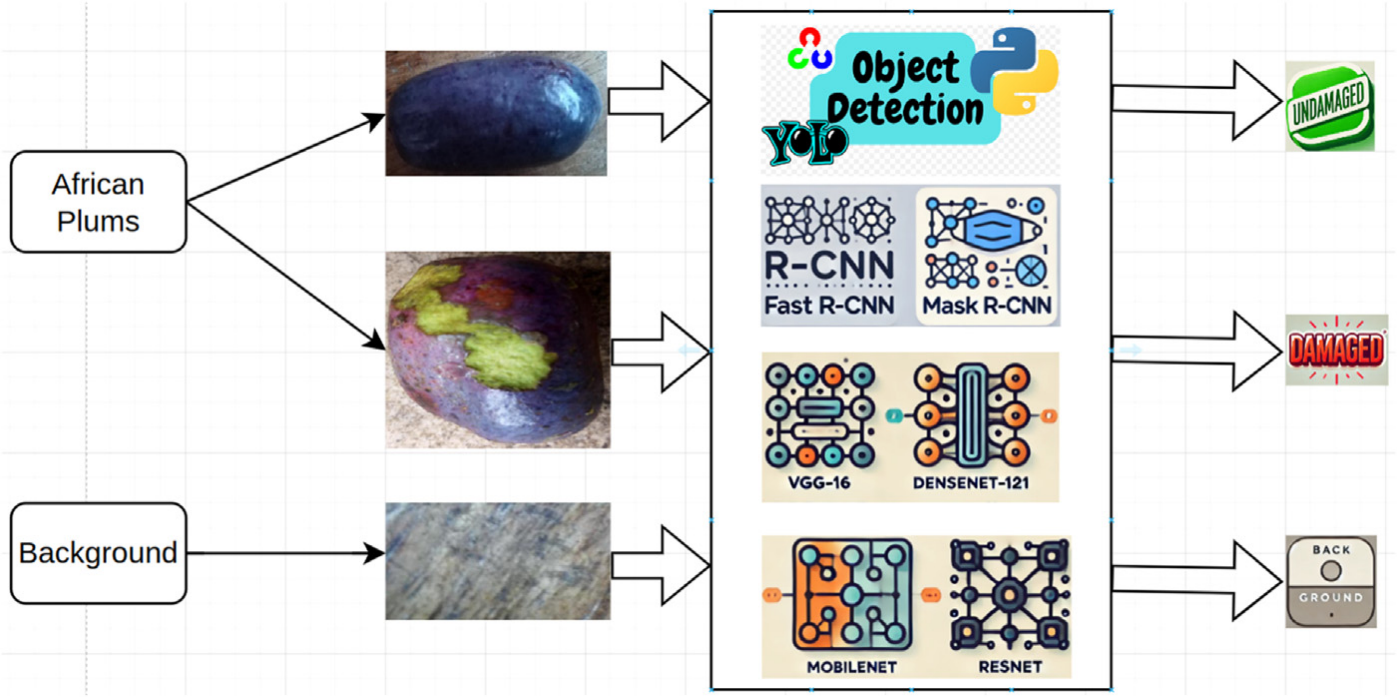
Plum classification in 3 categories, undamaged, damaged un background.

The dataset was utilized in the studies [2,3], where the authors introduce an intelligent vision system for real-time defect detection in African plums. The system classifies images into three categories, see Fig. 4: undamaged (i.e. unaffected plums), damaged (covering unripe, cracked, spotted, rotten, and bruised plums), and background (non-plum). Advanced models, including YOLOv5, YOLOv8, YOLOv9, Fast R-CNN, Mask R-CNN, VGG-16, DenseNet-121, MobileNet, and ResNet, were trained and pruned for efficiency. The results in the paper demonstrate that YOLOv8 model achieved the highest performance among object detection architectures with a mean average precision (mAP) of 93.6 %, while ResNet stood out among classification models with an accuracy of 90 % and an F1-score of 94 %. Pruning techniques applied to YOLO and ResNet models maintained high performance (e.g., YOLOv8 retained an mAP of 90.2 % with 10 % pruning), showcasing their potential for efficient deployment in real-time applications. In fact the highest-performing model, YOLOv8, was deployed, [19], in a web-based tool, offering farmers an accessible, AI-driven solution for plum quality assessment, thus improving agricultural productivity and quality control. A running instance [20]. These results highlight the models’ effectiveness in detecting and classifying surface defects in African plums, enabling practical use in agricultural quality assessment.

## 5 Limitations

While the dataset offers a substantial collection of images, its sample distribution is limited to specific regions in Cameroon where data collection was feasible. As a result, regional variations in plum appearance may not be fully represented. Additionally, environmental factors such as lighting and weather during image capture could introduce variability, affecting image consistency. Certain categories, notably cracked and bruised plums, are underrepresented, highlighting the need for further data collection to balance the dataset. Potential biases in image quality, due to fluctuating weather conditions and inconsistent daylight, should also be considered, as these factors may impact the performance of AI models trained on this dataset.Acknowledging these limitations helps users understand the dataset's scope and encourages further data augmentation or preprocessing steps to enhance model robustness.

## Ethics Statement

The authors confirm that this work does not involve human subjects, animal experiments, or any data collected from social media platforms.

## Credit Author Statement

**Arnaud Nguembang Fadja**: Conceptualization, Supervision, Data curation, Methodology, Writing – Original draft **Armel Gabin Fameni Tagni**: Data collection, Writing – Review & Editing **Sain Rigobert Che**: Data curation, Data labelling, Writing – Review & Editing **Marcellin Atemkeng**: Methodology, Writing – Review & Editing

## Conflict of Interest

The authors declare that they have no known competing financial interests or personal relationships that could have appeared to influence the work reported in this paper.

## Data Availability

KaggleAfrican Plums Dataset (Original data).
